# Response to ‘The evolving mystery of why skeletal muscle is spared in seropositive neuromyelitis optica’

**DOI:** 10.1111/jcmm.13513

**Published:** 2018-01-31

**Authors:** Antonio Frigeri, Grazia Paola Nicchia

**Affiliations:** ^1^ Department of Basic Medical Sciences, Neuroscience and Sense Organs University of Bari “Aldo Moro” Bari Italy; ^2^ Department of Bioscience, Biotechnology and Biopharmaceutics University of Bari “Aldo Moro” Bari Italy

Dear Editor,

We would like to thank you for the opportunity to respond to the questions raised in Dr Verkman's letter and to elucidate related aspects. We also thank Dr Verkman and colleagues for their attention to our study.

The use of g‐STED super‐resolution microscopy versus freeze‐fracture electron microscopy (FFEM) to analyse skeletal muscle and brain AQP4 supramolecular assemblies (OAPs) used in our study 1 has been disputed by Verkman *et al*. While we agree that FFEM is the gold standard to visualise OAPs and also measure their size, we also are aware that the very small amount of the plasma membrane that can be suitable for analysis represents a major limit to obtaining statistically significant data (such as the OAP dimension) representative of the entire tissue. In contrast, STED microscopy has the enormous advantage of analysing, in real time, very large portions of the plasma membrane, with a resolution that in our setup can reach approximately 30 nm, providing the possibility to have a more complete vision of the entire tissue and handle a large amount of data.

Considering the ‘contradiction of available data’, Verkman *et al*. refer to an FFEM study on OAP structure and organisation performed before the identification of AQP4 (or MIWC) as the molecular determinant of OAPs 2. Moreover, the same study did not directly compare muscle and brain OAP size 2. It was Verkman's group that later performed the first study 3 in which the role of AQP4 in OAP formation and in different tissues was directly analysed by FFEM in AQP4‐WT and null mice. This study literally reported: ‘The density of OAPs in brain was similar to that of OAPs in muscle, however, the patch sizes were somewhat bigger than in muscle…’ 3. Therefore, our interpretation is that our results are rather ‘in line with available data’ with a step forward in which g‐STED has helped to quantify the Verkman group's observation that OAPs in brain are ‘somewhat bigger that in muscle’. Anyhow, it is not of secondary importance that, independently of the size of skeletal muscle OAPs, super‐resolution microscopy revealed that AQP4 sarcolemma organisation in fast‐twitch skeletal muscle fibres is different compared to brain perivascular astrocyte endfeet.

A second issue raised by Verkman *et al*. refers to their own studies in which they have demonstrated that small changes in isoform ratio should not substantially affect NMO‐IgG binding 4. In this case, we have to take into account that those studies have two major limits: (1) they were obtained in heterologous systems in which only two isoforms were over‐expressed; and (2) they were obtained mainly using a recombinant monoclonal antibody, very far from the complexity of real human polyclonal autoantibodies, as demonstrated by epitope mapping studies 5. We believe that the unquestionable advantage of our study is that it has been performed on tissues expressing endogenous AQP4 with all the players (known and unknown) for AQP4 clustering. One of these players is the recently identified AQP4ex isoform 6, which is strongly expressed in skeletal muscle. As AQP4ex modulates AQP4 cluster size, 6 it may, for example, have a role in the different supramolecular organisation.

With regard to the concern about the use of non‐fixed frozen tissues in our study, it is well‐established that the use of unfixed tissue for immunofluorescence is crucial to preserve the conformational epitopes necessary for AQP4‐IgG binding 5.

While the fascinating mystery of why skeletal muscle is spared in seropositive neuromyelitis optica will certainly benefit from further studies, we believe that a very small piece has been added in this direction here.

We would like to thank all involved for the opportunity to continue this fascinating discussion and look forward to hearing from you.

Yours sincerely,

## Conflict of interest

The authors declare that they have no conflict of interest to disclose.
